# A scan statistic for continuous data based on the normal probability model

**DOI:** 10.1186/1476-072X-8-58

**Published:** 2009-10-20

**Authors:** Martin Kulldorff, Lan Huang, Kevin Konty

**Affiliations:** 1 Department of Population Medicine, Harvard Medical School and Harvard Pilgrim Health Care Institute, Boston, MA 02215, USA; 2 National Cancer Institute, Bethesda, MD, USA; Currently at the United States Food and Drug Administration, Rockville, MD, USA; 3 New York City Department of Health and Mental Hygiene, New York City, NY, USA

## Abstract

Temporal, spatial and space-time scan statistics are commonly used to detect and evaluate the statistical significance of temporal and/or geographical disease clusters, without any prior assumptions on the location, time period or size of those clusters. Scan statistics are mostly used for count data, such as disease incidence or mortality. Sometimes there is an interest in looking for clusters with respect to a continuous variable, such as lead levels in children or low birth weight. For such continuous data, we present a scan statistic where the likelihood is calculated using the the normal probability model. It may also be used for other distributions, while still maintaining the correct alpha level. In an application of the new method, we look for geographical clusters of low birth weight in New York City.

## Background

Spatial and space-time scan statistics [1-4] have become popular methods in disease surveillance for the detection of disease clusters, and they are also used in many other fields. In most applications to date, the interest has been in count data such as disease incidence, mortality or prevalence, for which a Poisson or Bernoulli distribution is used to model the random nature of the counts. For example, in papers published in 2008, Chen et al. [5] studied cervical cancer mortality in the United States; Osei and Duker [6] studied cholera prevalence in Ghana; Oeltmann et al. [7] looked at multidrug-resistant tuberculosis prevalence in Thailand; Mohebbi at al. [8] studied gastrointestinal cancer incidence in Iran; Rubinsky-Elefant et al. [9] looked at human toxocariasis prevalence in Brazil; Frossling et al. [10] evaluated the Neospora caninum distribution in dairy cattle in Sweden; Heres et al. [11] studied mad-cow disease in the Netherlands; and Reinhardt et al. [12] developed a system for prospective meningococcal disease incidence surveillance in Germany.

It is also of interest to detect spatial clusters of individuals or locations with high or low values of some continuous data attribute. Gay et al. [13] developed a spatial hazard model which they applied to detect geographical clusters of dietary cows with a high somatic cell score, which is a continuous marker for udder inflamation. Stoica et al. [14] has proposed a cluster detection method based on a number of random disks that jointly cover the cluster pattern in a marked point process. Huang [15] and Cook et al. [16] have developed spatial scan statistics for survival type data with censoring. The former applied the method to prostate cancer survival while the latter used their method for the time from birth until to asthma, allergic rhinitis or exczema. Other continuous data, such as birth weight [17] or blood lead levels, may be better modeled using a normal distribution, sometimes after a suitable transformation.

In this paper we develop a scan statistic for continuous data that is based on the normal probability model. Under the null hypothesis, all observations come from the same distribution. Under the alternative hypothesis, there is one cluster location where the observations have either a larger or smaller mean than outside that cluster. A key feature of the method is that the statistical inference is still valid even if the true distribution is not normal, assuring that the correct alpha level is maintained. This is accomplished by evaluating the statistical significance of clusters through a permutation based Monte Carlo hypothesis testing procedure. The new method is applied to birth weight data from New York City. A simulation study is performed to evaluate the power for different types of clusters.

The application and simulation results presented in this paper are concerned with two-dimensional spatial data, using a circular variable size scanning window. The new method is equally applicable to purely temporal and spatio-temporal data [18-20], to be used for daily prospective disease surveillance to look for suddenly emerging clusters. In addition to circles, it may also be used with an elliptic scanning window [21], or with any collection of non-parametric shapes [2,22-25].

The normal model has been incorporated into the freely available SaTScan software  for spatial and sdpace-time scan statistics, so it is easy to use. While it requires the use of computer intensive Monte Carlo simulations, computing times are very reasonable, unless the data set is huge.

## A Spatial Scan Statistic for Normal Data

### Observations and Locations

The data consists of a number of continuous observations, such as birth weight, with values *x*_*i*_, *i *= 1,...,*N*. Each observation is at a spatial location *s, s *= 1,...,*S*, with spatial latitude and longitude coordinates *lat*(*s*) and *long*(*s*). Each location has one or more observations, so that *S *≤ *N*.

For each location *s*, define the sum of the observed values as *x*_*s *_= ∑_*i*∈*s *_*x*_*i *_and the number of observations in the location as *n*_*s *_The sum of all the observed values are *X *= ∑_*i*_*x*_*i*_.

### Scanning Window

The circular spatial scan statistic is defined through a large number of overlapping circles [18]. For each circle *z*, a log likelihood ratio *LLR*(*z*) is calculated, and the test statistic is defined as the maximum LLR over all circles. The scanning window will depend on the application, but it is typical to define the window as all circles centered on an observation and with a radius varying continuously from zero up to some upper limit. To ensure that both small and large clusters can be found, the upper limit is often defined so that the circle contains at most 50 percent of all observations. It is never set above that number though, since a circular cluster with high values covering for example 80 percent of all observations is more appropriatly interpreted as a spatially disconnected 'cluster' with low values covering the 20 percent of observations that are located outside the circle, since it is those 20 percent that differ from the majority of observations. The maximum cluster size can also be defined using specific units of distance (e.g., 10 km). Circles with only one observation are ignored. Let *n*_*z *_= ∑_*s*∈*z*_*n*_*s *_be the number of observations in circle *z*, and let *x*_*z *_= ∑_*s*∈*z*_*x*_*s *_be the sum of the observed values in circle *z*.

### Likelihood Calculations

Under the null hypothesis, the maximum likelihood estimates of the mean and variance are *μ *= *X/N *and  respectively. The likelihood under the null hypothesis is then



and the log likelihood is



Under the alternative hypothesis, we first calculate the maximum likelihood estimators that are specific to each circle *z*, which is *μ*_*z *_= *x*_*z*_/*n*_*z *_for the mean inside the circle and *λ*_*z *_= (*X *- *x*_*z*_)/(*N *- *n*_*z*_) for the mean outside the circle. The maximum likelihood estimate for the common variance is



The log likelihood for circle *z *is



This simplifies to



As the test statistic we use the maximum likelihood ratio



or more conveniently, but equivalently, the maximum log likelihood ratio



Only the last term depends on *z*, so from this formula it can be seen that the most likely cluster selected is the one that minimizes the variance under the alternative hypothesis, which is intuitive.

### Randomization

The statistical significance of the most likely cluster is evaluated using Monte Carlo hypothesis testing [26]. Rather than generating random data from the normal distribution, a large set of random data sets are created by randomly permuting the observed values *x*_*i *_and their corresponding locations *s*. That is, the analysis is conditioned on the collection of continuous observations that were observed, as well as on the locations at which they were observed, which are considered non-random. By doing the randomization this way, the correct alpha level will be maintained even if the observations do not truly come from a normal distribution. Note that it is the individual observations that are permuted, so two different observations in the same location will end up in two different locations in most of the random data sets.

For each random data set, the log likelihood *lnL*(*z*) is calculated for each circle. The most likely cluster is then found and its log likelihood ratio is noted. If the log likelihood ratio from the real data set is among the 5 percent highest of all the data set, then the most likely cluster from the real data set is statistically significant at the 0.05 alpha level. More specifically, if there are *M *random data sets, then the p-value of the most likely cluster is *R*/(*M *+ 1), where *R *is the rank of the log likelihood ratio from the real data set in comparison with all data sets. In order to obtain nice p-values with a finite number of decimals, *M *should be chosen as for example 999, 4999 or 99999.

Note that these Monte Carlo based p-values are exact in the sense that under the null hypothesis, the probability of observing a p-value less than or equal to *p *is exactly *p *[26]. This is true irrespective of the number of random data sets *M*, but a higher *M *will provide higher statistical power.

If the random simulated data had instead been generated from a normal distribution with pre-specified mean and variance, rather than through permutation, then one would test the null hypothesis that the observations come from exactly that normal distribution. We would then reject the null for many reasons other than the existance of spatial clusters. For example, the null may be rejected because the mean values are higher than specified uniformly throughout the whole study region.

### Scanning for High or Low Values

As defined above, the normal scan statistic will search for clusters with exceptionally high values as well as clusters with exceptionally low values. Sometimes it makes more sense to only search for clusters with high values. The former is easily accomplished by adding an indicator function *I*(*μ*_*z *_>*λ*_*z*_) to the likelihood that is calculated under the alternative hypothesis. If one is only interested in cluster with low values, the indicator function is instead *I*(*μ*_*z *_<*λ*_*z*_).

### Software

The normal scan statistic has been incorporated into the freely available SaTScan™ software package, version 7.0 . It can be used for temporal, spatial and/or spatio-temporal data. The spatial version may be applied using a circular or elliptic window in two dimensions or a spheric window in three or more dimensions. The space-time version uses a cylindrical scanning window with either a circular or elliptic base. It is also possible for the user to define his/her own non-Euclidian neighborhood metric. The circle centroids can be identical to the collection of coordinates of the observations, or they may be specified by the user.

## Detection of Low Birth Weight and Early Gestation Clusters in New York City

The New York City Department of Health and Mental Hygiene (NYCDOH) calculates infant mortality rates by neighborhood and reports them in its annual Summary of Vital Statistics [27]. Though the infant mortality rate has fallen dramatically over the past 20 years (from 13.4 per 1000 in 1988 to 5.4 per 1000 in 2007) neighborhood variation can be quite high and this has attracted much attention from public health officials, the press, and researchers [28-30]. One approach to understanding the spatial pattern of infant mortality is to investigate the spatial patterns of known risk factors such as low birth weight, early gestation, or congenital conditions [28,17,32]. Attempts to identify clusters of low birth weight typically rely on dichotomized variables for birth weight (low: < 2500 grams; very-low: < 1500 grams) [17,31]. However, some researchers have noted a more complicated relationship among birth weight, gestation, and infant mortality with risk varying considerably within low birth weight and early gestation categories and modified by gender and other demographic characteristics [32].

This section examines spatial patterns of continuous measures of birth weight in New York City, using the spatial scan statistic with the normal probability model. Vital Records from NYCDOH were used to obtain data for all singleton births occurring in New York City in 2004. Births were geo-referenced to the Mother's zip code. Births to mother's not residing in New York City and those with invalid zips were deleted. Birth weight was measured in grams. The normal spatial scan statistic was used to detect clusters of low birth weight, using a circular window shape and 50 percent of all birth as the maximum cluster size.

**Figure 1:**
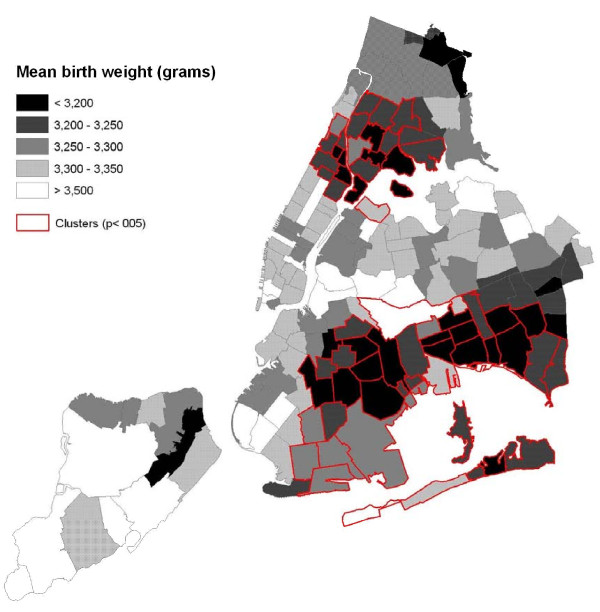
**The geographical distribution of birth weight in New York City zip codes in 2004, with two statistically significant clusters found by the spatial scan statistic with the normal probability model**.

Two statistically significant geographical clusters of low birth weight were found (Table 1 and Figure 1). With a log likelihood ratio (LLR) of 125.8, the first one consists of 61 zip-code areas in eastern Brooklyn and southern Queens, where the birth weights were on average 60 grams less than the rest of the city (*p *< 0.001). The second cluster consists of 29 zip-code areas in northern Manhattan and southern Bronx, where the birth weights were on average 52 grams less (*LLR *= 62.7, *p *< 0.001). The two statistically significant clusters correspond closely to areas of increased risk for infant mortality and are highly correlated with clusters found using dichotomized variables. There was also a non-significant single zip-code cluster on Staten Island (60 grams less, *LLR *= 3.6, *p *= 0.90). Note that, while the weight difference is as large or larger in the State Island cluster, such a difference could easily be due to chance, due to the small number of births inside the cluster. Note also that since a circular scanning window is used, some low birth weight areas are just outside the Brooklyn-Queens cluster while some high birth weigth areas are just inside. The key thing to realize is that it is only the general area of the cluster that is detected, not its exact boundaries.

**Table 1:** Geographical clusters of low birth weight in New York City in 2004.

	***Inside Cluster***		***Outside Cluster***		**Weight**	
**Cluster**	**#Births**	**Mean Weight (g)**	**#Births**	**Mean Weight (g)**	**Difference (g)**	**P-value**
Brooklyn/Queens	27772	3236	81152	3296	60	0.001
Manhattan/Bronx	16258	3236	92666	3288	52	0.001
Staten Island	617	3221	108307	3281	60	0.90

For the City as a whole, the variance of the birth weights is 297250 and the standard deviation is 545.2. After accounting for the different means inside and outside of the most likely cluster, the variance is 296564 and the standard deviation is 544.6. These number are, by default, lower, but only marginally so. In fact, the most likely cluster only explains (297250 - 296564)/297250 = 0.23 percent of the total variance. This is not surprising for an outcome such as birth weight, since the natural variation is rather large.

The spatial pattern of birth weight may be largely driven by the spatial patterns of demographic and pregnancy-specific characteristics. If so, clusters are not surprising; simply reflecting the geographical distribution of known characteristics. As such, the public health utility of clustering in the raw data may be limited. In a substantive paper, we hope to reexamine the geographical clusters of birth weight adjusting for the mother's demographics, health status, and pregnancy characteristics that are known to correspond with low birth weight. This has two uses: first, it sharpens understanding of the relationship between demographic covariates and birth weight and second, it identifies areas with surprisingly low birth weight for investigation, which cannot be explained in terms of their underlying demographics.

## Statistical Power and Spatial Precision

To evaluate the statistical power of the new method, we performed a simple simulation study. We simulated random normally distributed weights for infants born in New York City. The power to detect a cluster will depend on a number of factors, so data were generated using one standard baseline scenario and several variations: using different cluster locations within New York City, different cluster sizes, different sample size (total numbers of births), with different mean weights inside and outside the cluster, and with different variances inside and outside the true cluster.

In 2003, the average birth weight in New York City was around 3250 grams, with a standard deviation of approximately 600 grams. For a particular sample size, we fixed the total number of infants, and assigned them randomly to census tracts with the probability proportional to the census tract population size. All infants assigned into the same tract share the same latitude and longitude coordinates. This assignment was fixed and the same for all simulations with the same number of births.

**Figure 2:**
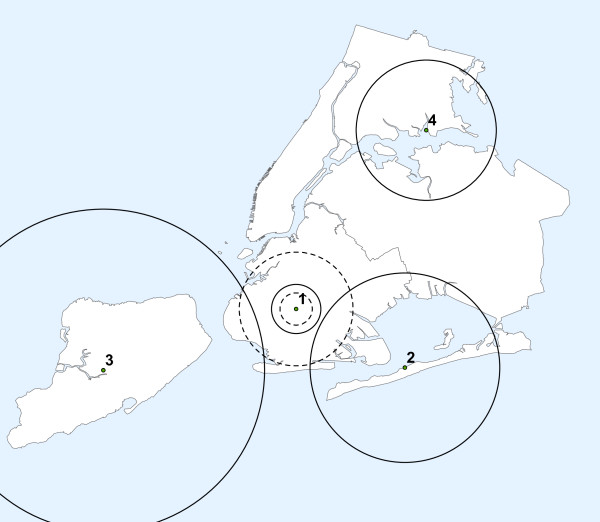
**The location and size of the artificial low birth weight clusters used to evaluate the statistical power of the spatial scan statistic for normally distributed data**.

We selected four cluster locations to have lower than average birth weight. These are shown in Figure 2. Cluster No. 1 is the baseline cluster, located in the center of Brooklyn. Cluster No. 2 is centered on The Rockaways, Queens, close to the Atlantic Ocean. Cluster No. 3 is located on Staten Island, far away from the rest of the City. Cluster No. 4 is split between southern Bronx and northern Queens. As the baseline, the maximum size of the clusters was defined to include 10 percent of all the births in the City. This means that the geographical size of the clusters varied, depending on the population density around the cluster centroid. For Cluster No. 1, we also evaluated clusters with 5 and 20 percent of the total number of births (dotted lines), while keeping the cluster centroid the same.

Let *Z *denote the collection of census tracts in the true cluster and let *Z*^*c *^denote the remaining New York City census tracts. For each of either 300, 600 (baseline), 900 or 1200 infants, we randomly simulate the birth weight from a normal distribution *N*(*μ*_*Z*_, *σ*^2^) if the infant was born inside the cluster and from *N*(, *σ*^2^) if the infant was born outside the cluster. For all simulations, we set  = 3250. We always choose *μ*_*Z *_<, so that the simulated data has a cluster of low birth weight. For the baseline, we chose *μ*_*Z *_to be ten percent less than , so that *μ*_*Z *_= 3250 - 325 = 2925. We also evaluated clusters where *μ*_*Z *_was 5, 8, 13, 15 and 20 percent less than . The variance *σ*^2 ^was the same inside and outside the cluster. For the baseline model we set the standard deviation to *σ *= 600, but we also evaluated *σ *= 300 and *σ *= 900. A complete list of all the evaluated cluster parameters are shown in the first five columns of Table 2.

**Table 2:** Estimated power, sensitivity and positive predictive value (PPV) when the normal scan statistic is used to detect different types of clusters, as described in the text.

**Cluster number**	**Cluster size (%)**	**Sample size**	**Cluster mean**	**Cluster STD**	**Power (%)**	**Sensitivity**	**PPV**
***Different Cluster Locations***							
1	10	600	-10%	600	66	0.70	0.74
2	10	600	-10%	600	54	0.56	0.65
3	10	600	-10%	600	64	0.72	0.82
4	10	600	-10%	600	61	0.61	0.64

***Different Cluster Size***							
1	5	600	-10%	600	34	0.52	0.48
1	10	600	-10%	600	66	0.70	0.74
1	20	600	-10%	600	96	0.87	0.89

***Different Sample Size***							
1	10	300	-10%	600	34	0.55	0.56
1	10	600	-10%	600	66	0.70	0.74
1	10	900	-10%	600	89	0.83	0.82
1	10	1200	-10%	600	98	0.85	0.85

***Different Mean Weight Reduction***							
1	10	600	-5%	600	14	0.30	0.38
1	10	600	-8%	600	42	0.56	0.61
1	10	600	-10%	600	66	0.70	0.74
1	10	600	-13%	600	92	0.84	0.84
1	10	600	-15%	600	98	0.88	0.88
1	10	600	-20%	600	100	0.93	0.93

***Different Standard Deviation***							
1	10	600	-10%	300	100	0.93	0.93
1	10	600	-10%	600	66	0.70	0.74
1	10	600	-10%	900	28	0.45	0.51

For each cluster scenario, we simulated 1000 random data sets. The estimated power is calculated as the proportion of the 1000 random data sets for which the null hypothesis was rejected, expressed as a percentage.

Even when the null hypothesis is correctly rejected, the detected cluster is usually not exactly identical to the true cluster. The extant of the overlap, and hence, of the spatial accuracy of the detected cluster, can be evaluated using sensitivity and positive predicted value (PPV). The sensitivity is de-fined as the proportion of the infants in the true cluster that was included in the detected cluster. This obviously varies between the random data sets, and the estimated sensitivity is taken as the average over the 1000 random data sets. The positive predictive value is defined as the proportion of the infants in the detected cluster that are in the true cluster. Again, this is estimated by taking the average over the 1000 random data sets.

The results are presented in Table 2. The power is approximately the same for the four different cluster locations, which is a reflection of the fact that they are about the same population size. As expected, the power increase when the cluster size increase, when the sample size increase, when the mean weight difference increase and when the standard deviation decrease. Sensitivity and positive predictive value follow the same pattern. Note that the sensitivity is about the same as the positive predictive value. This means that we are about equally likely to leave out an infant that should be in the cluster as we are to include an infant that shouldn't be in the cluster. Note also that even when the power is 100, the sensitivity and positive predictive value are not. This means that while we can determine the general location of a cluster, there will almost always be uncertainty when it comes to the borders of the detected cluster.

## Discussion

We have presented a scan statistic for continuous data. It is based on the normal distribution function, so if the data is truly normal, we have a likelihood ratio test. If the data follows some other distribution, it is no longer a likelihood ratio test, but it still maintains the correct alpha level. Hence, it can be used for a wide variety of continuous data, although we do not recommend it for exponential or other types of survival data, for which there are other scan statistics available [15,16].

The normal scan statistic performed well for the New York City birth weight data, finding two statistically significant clusters that corresponded to areas with high infant mortality.

The statistical power varies predictably with the type of cluster to be found. The same is true for sensitivity and the positive predictive value. One limitation of the simulation study is that we only evaluated the performance on data that were simulated from the normal distribution. While we know that the alpha level is correct for other distributions, we do not know about the power, sensitivity and positive predictive value.

As with most other scan statistics, the method is computer intensive, but not prohibitively so. The freely available SaTScan™ software  is available to do the calculations in a purely temporal, purely spatial or space-time setting, and when looking for clusters with either only high or only low values, or simultaneously for both. The normal probability model has been available in the SaTScan software since 2006, and the method has already been applied to study the epidemics of classical swine fever in Spain [33], the geographical differences in respondent and non-respondents in epidemiological studies [34] and the geographical clustering of the time people spend walking and bicycling in Los Angeles and San Diego [35]. The method can also be used in other fields outside of medicine and public health. For example, the variable of interest could be the amount of rainfall in various geographical locations in a country, pollution levels in a city, the height of plants on a field or the size of stars in a galaxy.

## Competing interests

The authors declare that they have no competing interests.

## Authors' contributions

MK obtained funding and developed the statistical methods. KK selected and performed the SaTScan analysis on the real data. MK and LH designed and LH performed the simulated power evaluations. MK, KK and LH wrote the first draft for different parts of the manuscript. All authors revised the manuscript and approved the final version.

## References

[B1] Naus J (1965). Clustering of random points in two dimensions. Biometrika.

[B2] Kulldorff M (1997). A spatial scan statistic. Communications in Statistics: Theory and Methods.

[B3] Glaz J, Balakrishnan N, editors (1999). Scan Statistics and Applications Birkäuser.

[B4] Glaz J, Naus J, Wallenstein S (2001). Scan Statistics.

[B5] Chen J, Roth RE, Naito AT, Lengerich EJ, MacEachren AM (2008). Geovi-sual analytics to enhance spatial scan statistic interpretation: An analysis of US cervical cancer mortality. International Journal of Health Geographics.

[B6] Osei FB, Duker AA (2008). Spatial dependency of V. cholera prevalence on open space refuse dumps in Kumasi, Ghana: A spatial statistical modeling. International Journal of Health Geographics.

[B7] Oeltmann JE, Varma JK, Ortega L, Liu Y, O'Rourke T, Cano M, Har-rington T, Toney S, Jones W, Karuchit S, Diem L, Rienthong D, Tappero JW, Ijaz K, Maloney S (2008). Multidrug-resistant tuberculosis outbreak among US-bound Hmong refugees, Thailand, 2005. Emerging Infectious Diseases.

[B8] Mohebbi M, Mahmoodi M, Wolfe R, Nourijelyani K, Mohammad K, Zeraati1 H, Fotouhi A (2008). Geographical spread of gastrointestinal tract cancer incidence in the Caspian Sea region of Iran: Spatial analysis of cancer registry data. BMC Cancer.

[B9] Rubinsky-Elefant G, Silva-Nunes M, Malafronte RS, Muniz PT, Ferreira MU (2008). Human toxocariasis in rural Brazilian Amazonia: Sero-prevalence, risk factors, and spatial distribution. American Journal of Tropical Medicine and Hygiene.

[B10] Frossling J, Nodtvedt A, Lindberg A, Björkman C (2008). Spatial analysis of Neospora caninum distribution in dairy cattle from Sweden. Geospa-tial Health.

[B11] Heres L, Brus DJ, Hagenaars TJ (2008). Spatial analysis of BSE cases in the Netherlands. BMC Veterinary Research.

[B12] Reinhardt M, Elias J, Albert J, Frosch M, Harmsen D, Vogel U (2008). EpiScanGIS: An online geographic surveillance system for meningococcal disease. International Journal of Health Geographics.

[B13] Gay E, Senoussi R, Barnouin J (2007). A spatial hazard model for cluster detection on continuous indicators of disease: Application to somatic cell score. Veterinary Research.

[B14] Stoica RS, Gay E, Kretzschmar A (2007). Cluster pattern detection in spatial data based on Monte Carlo inference. Biometrical Journal.

[B15] Huang L, Kulldorff M, Gregorio D (2007). A spatial scan statistic for survival data. Biometrics.

[B16] Cook A, Gold DR, Li Y (2007). Spatial cluster detection for censored outcome data. Biometrics.

[B17] Ozdenerol E, Williams BL, Kang SY, Magsumbol MS (2005). Comparison of spatial scan statistic and spatial filtering in estimating low birth weight clusters. International Journal of Health Geographics.

[B18] Kulldorff M, Athas W, Feuer E, Miller B, Key C (1998). Evaluating cluster alarms: A space-time scan statistics and brain cancer in Los Alamos. American Journal of Public Health.

[B19] Kulldorff M (2001). Prospective time-periodic geographical disease surveillance using a scan statistic. Journal of the Royal Statistical Society Series A.

[B20] Kulldorff M, Heffernan R, Hartman J, Assunção R, Mostashari F (2005). A space-time permutation scan statistic for the early detection of disease outbreaks. PLoS Medicine.

[B21] Kulldorff M, Huang L, Pickle L, Duczmal L (2006). An elliptic spatial scan statistic. Statistics in Medicine.

[B22] Patil GP, Taillie C (2003). Geographic and network surveillance via scan statistics for critical area detection. Statistical Science.

[B23] Duczmal L, Assunçao R (2004). A simulated annealing strategy for the detection of arbitrarily shaped spatial clusters. Computational Statistics and Data Analysis.

[B24] Tango T, Takahashi K (2005). A flexibly shaped spatial scan statistic for detecting clusters. International Journal of Health Geographics.

[B25] Assunçao RM, Costa M, Tavares A, Ferreira S (2006). Fast detection of arbitrarily shaped disease clusters. Statistics in Medicine.

[B26] Dwass M (1957). Modified randomization tests for nonparametric hypotheses. Annals of Mathematical Statistics.

[B27] Office of Vital Statistics (2004). Summary of Vital Statistics 2004, the City of New York New York.

[B28] Sohler NL, Arno PS, Chang CJ, Fang J, Schechter C (2003). Income inequality and infant mortality in New York City. Urban Health.

[B29] Grady SC, McLafferty S (2007). Disentangling the effects of residential segregation and neighborhood poverty on low birthweight for immigrant and native-born black women in New York City. Urban Geography.

[B30] Grady SC (2006). Racial disparities in low birthweight and the contribution of residential segregation: A multilevel analysis. Social Science and Medicine.

[B31] Rushton G, Krishnamurthy R, Krishnamurt R, Lolonis P, Song H (1996). The spatial relationship between infant mortality and birth defect rates in a U.S. city. Statistics in Medicine.

[B32] Solis P, Pullman SG, Frisbie WP (2000). Demographic models of birth outcomes and infant mortality: An alternative measurement approach. Demography.

[B33] Martínez-López B, Perez AM, Sánchez-Vizcaíno JM (2009). A stochastic model to quantify the risk of introduction of classical swine fever virus through import of domestic and wild boars. Epidemiology and Infection.

[B34] Shen M, Cozen W, Huang L, Colt J, De Roos AJ, Severson RK, Cerhan JR, Bernstein L, Morton LM, Pickle L, Ward MH (2008). Census and geographic differences between respondents and nonrespondents in a case-control study of non-Hodgkin lymphoma. American Journal of Preventive Medicine.

[B35] Huang L, Stinchcomb D, Pickle L, Dill J, Berrigan D (2009). Identifying clusters of active transportation using spatial scan statistics. American Journal of Preventive Medicine.

